# Supra-aortic bypass reconstructions using ring-reinforced 8 mm polytetrafluoroethylene for debranching before thoracic endovascular aortic repair and for atherosclerotic lesions of the subclavian artery

**DOI:** 10.1016/j.jvscit.2026.102406

**Published:** 2026-07-11

**Authors:** Mikolaj Walensi, Kai Nassenstein, Theresia Knop, Susanne B. Kiewitz, Nikolaos Tsilimparis, Eike S. Debus, Michal Piotrowski, Nikodem Ulatowski, Gundula Schulz, Johannes N. Hoffmann

**Affiliations:** a Department of Vascular Surgery and Phlebology, Elisabeth-Hospital Essen, CONTILIA Group, Heart- and Vascular Center, affiliated with the University Hospital of Essen, Essen, Germany; b Department of Vascular Surgery, University Hospital LMU Munich, Munich, Germany; c Department of Diagnostic and Interventional Radiology and Neuroradiology, University Hospital Essen, Essen, Germany; d Department of Vascular Medicine, University Heart and Vascular Center UKE Hamburg, University Medical Center Hamburg-Eppendorf, Hamburg, Germany; e Department of Emergency Medicine, McMaster University, Hamilton, Ontario, Canada; f Department of Cardiac and Vascular Surgery, Medical University of Gdansk, Gdansk, Poland

**Keywords:** Carotid-subclavian bypass, Carotid-carotid bypass, Debranching, 8 mm, Polytetrafluoroethylene

## Abstract

Numerous cardiovascular pathologies, including thoracic endovascular aortic repair, may require supra-aortic bypass (SB) reconstruction. This study reports the safety and efficacy of SB reconstructions using ring-reinforced 8 mm polytetrafluoroethylene. From April 2017 to June 2023, 17 patients (9 female; median age, 72 years) underwent creation of 23 SBs. Indications included thoracic endovascular aortic repair debranching in 10 patients and symptomatic subclavian artery stenosis or occlusion in 7 patients. No intraoperative complications, bypass revisions, or 30-day mortality occurred. After follow-up of up to 5.9 years, one bypass occlusion was observed; all examined patients were asymptomatic and 19 bypasses (96%) remained patent, demonstrating excellent feasibility, safety, and patency.

Numerous vascular pathologies require the creation of a supra-aortic bypass (SB).^1^, ^2^, ^3^, ^4^, ^5^ Regarding the materials and its specifications used, for example, carotid-subclavian bypass (CSB) or carotid-carotid bypass (CCB), the evidence is scarce. Although venous bypasses are described to have poor patency rates and their use should be restricted to particular indications,^6^, ^7^, ^8^, ^9^ polyethylene terephthalate (PET, Dacron; DuPont) and, later, polytetrafluoroethylene (PTFE, Gore-Tex; W. L. Gore & Associates) have been used for SB creation.^7^, ^8^, ^9^, ^10^, ^11^ The use of prosthetic bypass materials has been established and studied quite thoroughly for peripheral bypasses in patients with peripheral arterial occlusive disease (PAOD) of the lower limb. There is some research comparing PET and PTFE^12^, ^13^, ^14^, ^15^; some of it suggests PTFE to provide higher patency rates and being associated with lower morbidity rates.^16^ With regard to SB reconstructions, there are far less publications which investigate the properties of different bypass materials for SB creations. Regarding certain PTFE specifications (ie, diameter and ring-reinforcement), due to the lack of standardization even less data is available for the various options when this material is used.^9^^,^^17^, ^18^, ^19^ The technical, intraoperative, perioperative, and follow-up results are presented to demonstrate that the standardized use of ring-reinforced 8 mm PTFE SB provides excellent patency rates and a high safety profile, particularly without ring-related complications in the cervical region.

## Methods

**Fig 1:**
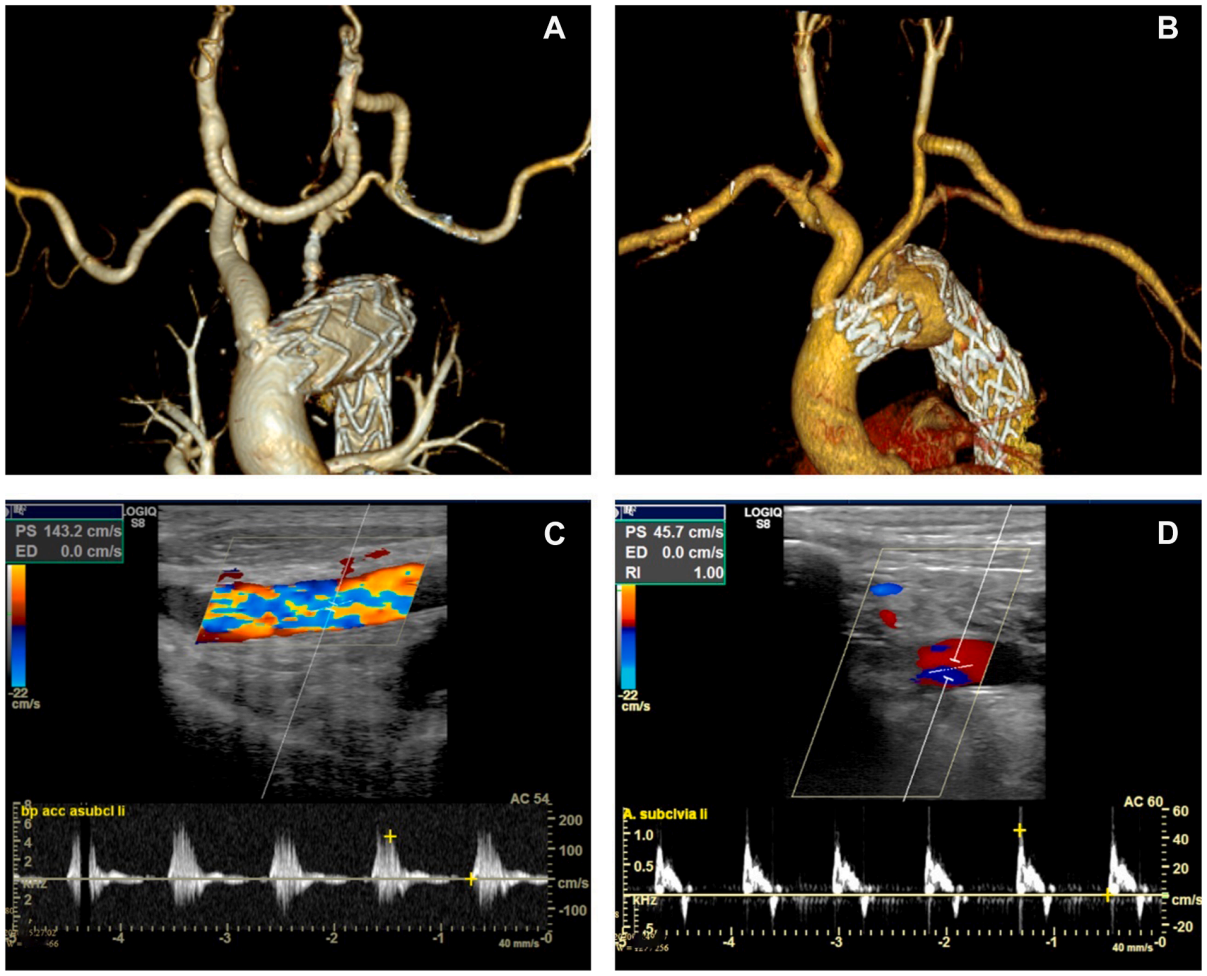
**A,** Three-dimensional (3D)-reconstruction of a computed tomography (CT)-angiography of a supra-aortic reconstruction with a ring-reinforced 8 mm polytetrafluoroethylene (PTFE) carotid-subclavian and a carotid-carotid bypass in a 78-year-old male patient with an aortic arch aneurysm who required endovascular repair. This CT-angiography has been performed 6 years and 3 months after the bypass reconstructions, currently showing no evidence of an anastomotic stenosis or bypass occlusion. **B,** 3D-reconstruction of a CT-angiography of a supra-aortic reconstruction with a ring-reinforced 8 mm PTFE carotid-subclavian bypass in a 78-year-old male patient with an aortic arch aneurysm. The CT-angiography has been performed 3 months after the bypass reconstructions, currently showing no evidence of an anastomotic stenosis or bypass occlusion. **C,** Triplex sonography of a supra-aortic reconstruction with a ring-reinforced 8 mm PTFE carotid-subclavian in a 52-year-old female patient with a thoracic aortic aneurysm and an aneurysm of the aortic arch, showing the carotid-subclavian bypass with a triphasic Doppler waveform and a flow of 1.43 m/s in the bypass. **D,** Triplex sonography of a supra-aortic reconstruction with a ring-reinforced 8 mm PTFE carotid-subclavian in a 52-year-old female patient with a thoracic aortic aneurysm and an aneurysm of the aortic arch, showing the restored perfusion of the subclavian artery after the arterial reconstruction with a triphasic Doppler waveform and a flow of 0.46 m/s in the distal subclavian artery.

The data of all patients who underwent SB creation using ring-reinforced 8 mm PTFE grafts were collected concurrently with daily clinical documentation, whereas final analysis was performed retrospectively after completion of all procedures. Patients' demographics, risk factors, indication for surgery, clinical presentation, intraoperative and perioperative data and complications, as well as hospitalization time and follow-up data were analyzed with special attention paid to the patency and perfusion of the SB grafts. According to current national and international guidelines for quality control in carotid surgery, SB patency has been proven by intraoperative completion angiography, duplex sonography, and flow measurement (Transonic Clinical Flowprobes, Transonic Systems Inc) as well as repetitive duplex ultrasound study and/or computed tomography angiography (Fig 1).^20^, ^21^, ^22^ Perfusion has been ascertained by clinical examination and Doppler occlusion pressure measurements. Brachial blood pressure was measured manually using a standard sphygmomanometer and cuff (Baxter International, Hillrom, Welch Allyn, FlexiPort blood pressure cuff, Adult 11, 34-35 cm) and a Doppler device (ELCAT GmbH, handydop, 5 MhZ probe). The indication for the procedure was established interdisciplinary during a triple-certified vascular board meeting involving specialists from radiology, angiology, and vascular surgery. A neurological specialist performed a neurological examination before and after surgery. On the first postoperative day, a clinical and neurological examination was performed, along with a Doppler pressure measurement and a duplex ultrasound study of the bypass and the supra-aortic vessels. Follow-up examinations were performed in the outpatient vascular clinic at 6 weeks as well as 3, 6, and 12 months postoperatively, followed by subsequent annual investigations in case of an uneventful course. Due to the observational study design, an ethical approval was not required.

### Surgical procedure

**Fig 2:**
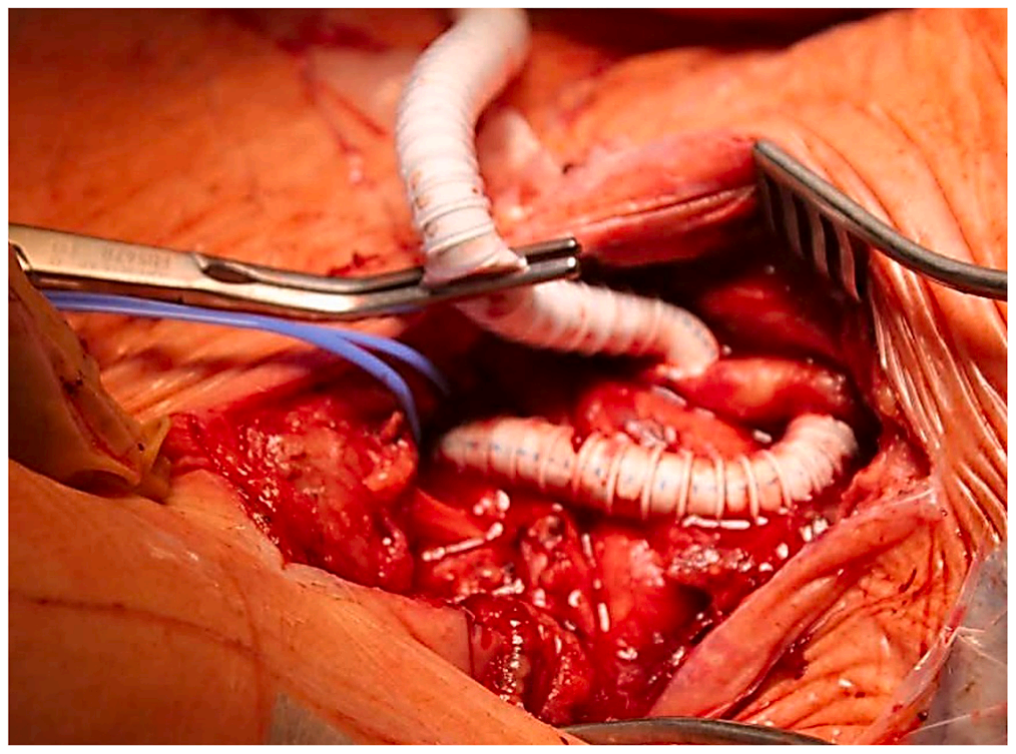
Intraoperative view of an already created ring-reinforced 8 mm polytetrafluoroethylene (PTFE) carotid-subclavian bypass and the anastomosis to the left common carotid artery of the upcoming ring-reinforced 8 mm PTFE carotid-carotid bypass in a 68-year-old male patient receiving a debranching procedure before thoracic endovascular aortic repair (TEVAR). Note the lateral (side-to-end) bypass insertion of the carotid-subclavian bypass into the left common carotid artery and the medial (side-to-end) insertion of the carotid-carotid bypass into the left common carotid artery. Pretracheal tunneling of the carotid-carotid bypass will subsequently be performed directly posterior of the jugular notch. There were no complications at the surgical site noted in any of our patients.

All procedures were performed under general anesthesia using single shot antibiotic prophylaxis with cefuroxime 1500 mg intravenously. The patient was placed in a supine position with the torso slightly elevated. For CSB, an incision was made above the clavicle, extending to the posterior edge of the sternocleidomastoid muscle to expose the subclavian artery via a supraclavicular lateral approach. The common carotid artery was exposed through a dorsal approach from the same incision. Heparin sodium (5,000 IU, intravenously) was administered before clamping of the arteries. First, the distal anastomosis of the ring-reinforced 8 mm PTFE bypass with the subclavian artery was created using a 6-0 monophilic continuous suture in end-to-side-manner. After examination of the tightness of the anastomosis, the bypass graft was tunneled through the deep cervical space, posterior to the left internal jugular vein and deep to the platysma, pulled toward the common carotid artery. The bypass was shortened while retaining a modest amount of length redundancy to allow for physiological movement without tension^23^ and a proximal side-to-end-anastomosis was created using a 6-0 monophilic continuous suture. After completion of the bypass, blood flow was restored – first into the arm, followed by the subclavian artery and finally the common carotid artery to prevent cerebral embolization. For supplementary CCB creation (Fig 2), an additional incision on the right neck was performed, and the common carotid artery was exposed at the posterior edge of the right sternocleidomastoid muscle at the level of the omohyoid muscle. The CCB graft was anastomosed end-to-side to the left common carotid artery, tunneled pretracheally (collar technique, preferred over a retrotracheal approach due to a less invasive surgical dissection, a reduced risk of nerve and esophageal injury, and additionally straightforward accessibility for postoperative duplex ultrasound surveillance) and anastomosed side-to-end to the right common carotid artery, each time using a 6-0 monophilic continuous suture. In both SB variants, the affected supra-aortic artery was ligated strictly proximal to the origin of the vertebral artery after bypass creation. Shunting was not performed in either SB creation. Redon suction drains were placed at all incisions and systematically removed 1 to 2 days postoperatively. Perioperatively, patients received unfractionated heparin and later low-molecular-weight heparin for thrombosis prophylaxis unless there was an indication for therapeutic anticoagulant therapy. Patients were discharged with at least one single antiplatelet agent.

### Statistics

Prospective data acquisition and subsequent retrospective analysis were performed. Data collection was carried out using Excel (Microsoft), and statistical analysis was conducted using SPSS version 29.0 (IBM Corp). The normality of the differences was assessed using the Shapiro-Wilk test. If the assumption of normality was met, a paired *t* test was performed; otherwise, the Wilcoxon signed-rank test was applied. A *P* value of <.05 was considered statistically significant. All values are given as n (%) or median (range).

## Results

From April 2017 to June 2023, 17 consecutive patients [9 female (53%)] underwent a total of 23 SB creations using ring-reinforced 8 mm PTFE grafts, all in an (semi-)elective setting. Patients' characteristics and demographics are given in Table I. Indications for surgery were debranching procedures before thoracic endovascular aortic repair (TEVAR) in 10 patients (59%) [2 patients (12%) with a penetrating ulcer of the aorta, 2 patients (12%) with a combined aortic arch and thoracic aortic aneurysm, 2 patients (12%) with a thoracic aortic aneurysm, 2 patients (12%) with a thoracoabdominal aneurysm, 1 patient (6%) with an isolated aortic arch aneurysm, 1 patient (6%) with a non-A-non-B-dissection], and bypass creation due to a symptomatic subclavian artery stenosis (1) or occlusion (6) in 7 patients (41%). Of the 23 SB, 17 (74%) were CSB and 6 (26%) were CCB (always performed simultaneously with a CSB as part of a debranching procedure before TEVAR). Of the 10 TEVAR procedures, 3 (18%) were performed during the same hospital stay as the debranching procedure.

**Table I:** Patients' characteristics and demographics

Patients' data	
Age, years	72 (52-88)
BMI, kg/m^2^	26.1 (21.1-35.5)
ASA score	3 (2-4)
Comorbidities	
Coronary heart disease	3 (18)
Arterial hypertension	13 (76)
Chronic obstructive pulmonary disease	3 (18)
Hyperlipoproteinemia	8 (47)
Renal impairment	6 (35)
Diabetes mellitus	3 (18)
Smoking	3 (18)
Antithrombotic and anticoagulative drugs	
Acetylsalicylic acid	12 (71)
Clopidogrel	2 (12)
Phenprocoumon	2 (12)
DOAC	2 (12)

*ASA*, American Society of Anesthesiologists; *BMI*, body mass index; *DOAC*, direct oral anticoagulants.

Categorical data are shown as number (%).

Intraoperative and perioperative data are given in Table II. The creatinine values (in mg/dL) were 0.87 (0.67-2.66) preoperatively and 0.73 (0.55-1.83) postoperatively (*P* = .002), whereas the hemoglobin levels (in g/dL) were 14.1 (9.7-16.6) preoperatively and 11.4 (7.0-13.6) postoperatively (*P* ≤.01), respectively. No intraoperative complications occurred, and no intraoperative bypass revision was required. The intraoperatively measured bypass flow was 255 (60-360) mL/min.

**Table II:** Intraoperative and perioperative data

Operation time, minutes	122 (85-213)
Combined (carotid-carotid and carotid-subclavian)	191 (160-213)
Solitary (carotid-subclavian)	111 (85-132)
Clamping time, minutes	
Combined (carotid-carotid and carotid-subclavian)	28 (23-43)
Left CCA	18 (11-31)
Right CCA	10 (6-12)
Solitary (carotid-subclavian, left CCA)	14 (10-28)
Blood loss, mL	200 (100-700)
Combined (carotid-carotid and carotid-subclavian)	500 (100-700)
Solitary (carotid-subclavian)	100 (100-400)
Packed red blood cells transfusion	2 (12)
Intraoperatively	2 (12)
No. transfused	0 (0-3)
Postoperatively	0 (0%)
No. transfused	0 (0-0)
Hospitalization time, days	
Solitary bypass procedures	10 (6-18)
Bypass procedures and TEVAR combined (same stay)	12 (6-24)
Time from bypass procedure until TEVAR (same stay)	3 (1-10)

*CCA*, Common carotid artery; *TEVAR*, thoracic endovascular aneurysm repair.

All values are given as n (%) or median (range).

Regarding postoperative complications, in-hospital- and 30-day-mortality and stroke rates and bypass closure rates were all zero. Early postoperative complications were as follows: 3 (13%) cervical hematomas [1 (4%) requiring surgical revision in a patient with giant cell arteritis and severe large vessel disease, 1 (4%) prolonged cervical wound healing, and 1 (4%) hyponatremia] and 2 (9%) hypertensive urgencies. Information regarding postoperative antiplatelet and anticoagulation therapy at discharge is given in Table I.

**Fig 3:**
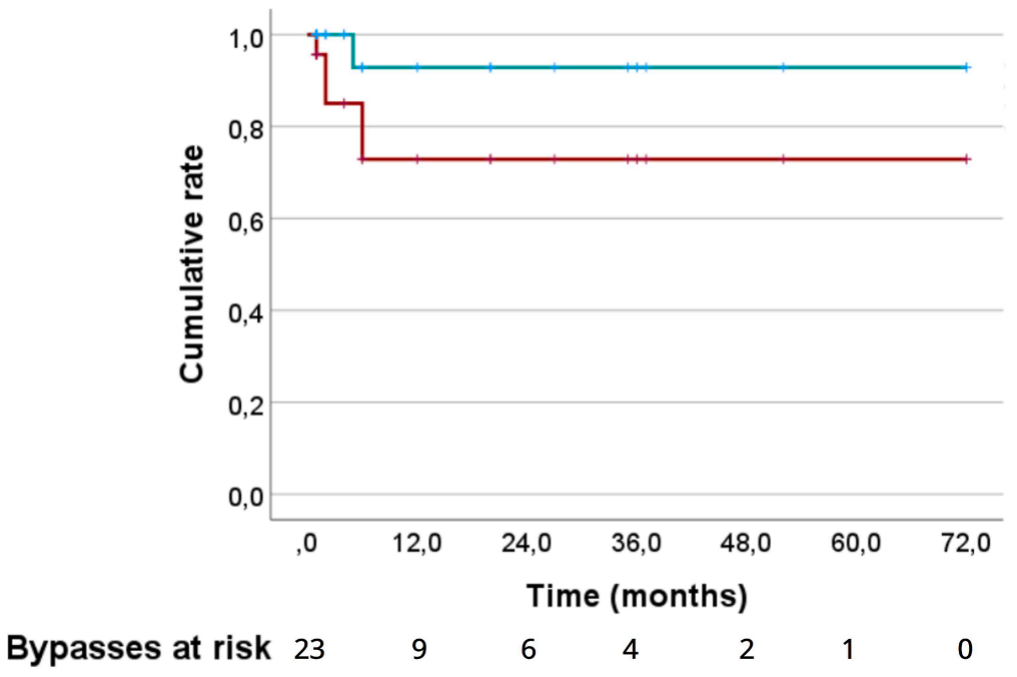
Kaplan-Meier curves demonstrating cumulative primary patency rates (*blue line*) and overall survival rates (*red line*) over a 72-month follow-up period. Vertical ticks indicate censored observations. The number of bypasses at risk for each time interval is provided in the table below the horizontal axis.

Regarding long-term morbidity and mortality, 1 patient (4%) suffered from an occlusion of the CCB 5 months after surgery (treated with a thrombectomy) and died of pneumonia 6 months after the debranching procedure. Two patients (9%) died after the TEVAR procedure [1 patient (4%) of fatal myocardial infarction 2 months after the debranching procedure and 1 patient (4%) of fatal pulmonary embolism 1 month after the debranching procedure]. One (4%) patient suffered from a lymphatic drainage obstruction at the bypass site 12 months after surgery with edema of the arm, which was treated conservatively using compression therapy and manual lymphatic drainage. No patient suffered from an arm or hand claudication or coolness of the limb of the operated site. No stroke events, internal jugular vein thromboses, phrenic, or recurrent laryngeal nerve injuries occurred. There was no statistically significant difference between either the systolic and diastolic blood pressure values nor between the measurements from the radial and ulnar arteries on the operated side. Both were compared with the immediate postoperative values and in follow-up examinations between the operated and nonoperated sides (Tables III). Long-term postoperative primary patency rates of the bypasses were 23 (100%) after 6 weeks (3-9 weeks), 21 (100%) after 3 months (2-4 months), 19 (96%) after 6 months (5-10 months), and 19 (96%) after 12 months (8-22 months). Secondary patency rate was 100% across all follow-up intervals due to the one aforementioned thrombectomy. After the maximal median follow-up period of 3 years (1.6-5.9 years), all 14 examined patients (3/17 died) were asymptomatic and all 19 (96%) bypasses were patent. Kaplan-Meier curves illustrating bypass graft patency and survival are given in Fig 3.

**Table III:** Occlusion pressure and blood pressure measurements (mmHg, median, range, and %) of the operated side over time (A), and comparison between operated and contralateral side at follow-up time points (B).

(A)	Immediately after SB surgery	6 months after SB surgery	*P* value	12 months after SB surgery	*P* value
Radial artery, mmHg	130 (120-140)	140 (120-160)	.43	140 (120-170)	.18
Ulnar artery, mmHg	130 (120-140)	140 (120-160)	.34	140 (110-170)	.46
Brachial artery, systolic, mmHg	120 (110-140)	140 (110-160)	.15	140 (140-150)	.10
Brachial artery, diastolic, mmHg	80 (70-90)	75 (70-90)	>.99	75 (70-80)	.18

(B)	Immediately after SB surgery operated site	Immediately after SB surgery contralateral site	*P* value
Radial artery	130 (120-140)	130 (120-140)	.67
Ulnar artery	130 (120-140)	130 (120-140)	.32
Brachial artery, systolic	120 (110-140)	120 (110-140)	.68
Brachial artery, diastolic	80 (70-90)	80 (70-90)	.67

	6 months after SB surgery operated site	6 months after SB surgery contralateral site	*P* value
Radial artery	140 (120-160)	140 (110-160)	.37
Ulnar artery	140 (120-160)	140 (90-160)	.37
Brachial artery, systolic	140 (110-160)	140 (110-160)	>.99
Brachial artery, diastolic	75 (70-90)	75 (60-90)	.32

	12 months after SB surgery operated site	12 months after SB surgery contralateral site	*P* value
Radial artery	140 (120-170)	150 (120-180)	.16
Ulnar artery	140 (110-170)	150 (120-180)	.10
Brachial artery, systolic	140 (140-150)	140 (140-150)	>.99
Brachial artery, diastolic	75 (70-80)	75 (70-80)	>.99

*SB*, Supra-aortic bypass.

## Discussion

In this patient population, the use of ring-reinforced 8 mm PTFE grafts for CSB and CCB resulted in excellent perioperative safety and durable long-term graft patency, with serial hemodynamic assessments revealing no significant differences in occlusion pressures or limb blood pressures between treated and contralateral sides throughout follow-up.

Certain pathologies require bypass procedures for revascularization of blood flow in the supra-aortic arteries, especially the subclavian and carotid artery.^1^, ^2^, ^3^, ^4^, ^5^ Although the first surgical techniques reported by Crawford et al^24^ used a transthoracic approach for bypass grafting and showed high mortality rates,^7^^,^^25^ the CSB as a less invasive method has been introduced by Diethrich et al.^26^ CSB, subclavian-carotid, and CCB reconstructions have meanwhile become well established and common surgical procedures to treat symptomatic stenotic or occlusive diseases of the supra-aortic branches.^7^^,^^9^^,^^27^ Also, patients who need to undergo TEVAR for thoracic or thoracoabdominal aortic aneurysms may require debranching via these bypasses to create a sufficient landing zone for the upcoming stent graft in the Ishimura zone 2 of the aortic arch.^28^ Given that endovascular or hybrid treatment for thoracic and thoracoabdominal aortic aneurysms often yield superior outcomes compared with open surgical repair, achieving success depends on a sufficient debranching procedure. Using an optimal graft setup is crucial for therapeutic success; however, this remains a technique of last resort in the endovascular era.^2^^,^^29^, ^30^, ^31^, ^32^, ^33^, ^34^ Also, even rare disorders like the coronary-subclavian steal syndrome may be treated using a CSB as the segment of the subclavian artery between its origin and the left internal mammary artery has become part of the coronary circulation after aortocoronary artery bypassing.^5^^,^^8^^,^^27^^,^^35^, ^36^, ^37^, ^38^ Regarding these particular indications and techniques, the optimal material for the SB reconstructions is still being discussed.^7^, ^8^, ^9^, ^10^, ^11^ Based on evidence from the literature, ring-reinforced 8 mm PTFE has been used as the material of choice for SB creation.

However, only few publications can be found investigating the properties of different bypass materials for SB creations. Even less data is available on the features of certain PTFE specifications like the bypass diameter or the presence of ring-reinforcement.^10^^,^^17^^,^^19^^,^^39^

Regarding the specification of ring-reinforcement in PTFE grafts, larger series of SB creations such as Byrne et al,^11^ Cinar et al,^27^ or Abu Rahma et al^40^ report the use of 6 to 8 mm PTFE grafts, whereas there is no information about the presence of ring-reinforcement at all. Here, patency rates after 5 years reached 92%, 83%, and 96%, respectively. Although these authors do not mention ring-reinforcement, Illuminati et al^39^ reached a 5-year-patency rate of 96% by using ring-reinforced 7 mm PTFE, whereas Gupta et al^17^ reached a 5-year-patency rate of 92% using ring-reinforced 6 mm PTFE, respectively, which might indicate better patency rates for greater diameters. Also, Owens et al^41^ and Gupta et al^17^ advocate for ring-reinforced PTFE, emphasizing its greater durability and resistance to compression in SB reconstructions. In a small case series, Ochi et al^42^ used ring-reinforced 8 mm PTFE for simultaneous subclavian artery reconstruction during coronary artery bypass grafting, reporting complication-free courses and sustained patency for up to 50 months. Although there are no specific investigations on ring reinforcement in studies on SBs, some studies on lower-extremity revascularization suggest an advantage for ring-reinforced bypasses. For infrapopliteal bypass reconstructions, Müller-Wiefel^43^ reported the standardized use of ring-reinforced PTFE grafts, achieving an 81% patency rate in patients with good run-off conditions. Moreover, Kashyap et al^44^ demonstrated a significantly higher patency rate for ring-reinforced PTFE grafts (65%) compared with nonringed grafts (40%). Furthermore, Samson et al^45^ used ring-reinforced 8 mm PTFE grafts as the standard configuration for creating axillounilateral and axillobilateral bypasses, and thus for long-segment and complex revascularizations in patients with PAOD, achieving a 5-year patency rate of up to 84%. With regard to the diameter, Vitti et al^9^ and Voigt et al^19^ reached patency rates of 93% after 4.25 years and 97% after 5 years respectively by using 8 mm PTFE grafts; however, both do not mention the use of ring-reinforcement. Interestingly, Wittwer et al^10^ found a tendency toward a better 5-year patency rate (93% vs 79%) for 8 mm grafts compared with 6 mm grafts, irrespective to the graft material, also suggesting a better performance of larger diameters. Accordingly, Hepp et al^46^ described a higher patency rate for 8 mm compared with 6 mm PET grafts.

Regarding the bypass materials, PTFE has been compared with PET, as well as to autologous vein grafts. In contrast to bypasses of the lower leg in patients with PAOD, autologous vein grafts demonstrated in some studies significantly worse performance compared with PTFE and PET.^6^, ^7^, ^8^, ^9^, ^10^ This is very likely due to its higher vulnerability to kinking from neck motion and its smaller diameter resulting in poorer long-term patency rates.^7^^,^^39^ Being more resistant to infections, autologous vein grafts should therefor only be considered for the treatment of bypass infections or surgery in septic patients as they have no evident benefit over prosthetic material in standard situations.^7^^,^^39^ At the same time, the inherent stiffness of prosthetic graft materials may provide advantages for the bypass creation, especially in this position as it may prevent kinking and bypass occlusion while being adjusted for tension.^7^ Also, especially PTFE provides additional features such as low thrombogenicity, rapid and reliable incorporation, resistance to infection and perigraft reactions, as well as the longevity of the material.^18^^,^^39^ Especially for perigraft reactions literature shows relatively high rates of lymphatic complications for PET grafts. Here, Mehmedovic et al^47^ showed that PET grafts have a 2.4% incidence of seroma, whereas Konstantinou et al^48^ reported a 4% occurrence of chylous fistula and Ince et al^49^ reported lymphorrhea in 10%, all in debranching procedures before TEVAR. A recent study by Mehra et al^50^ even reports a lymph fistula rate of 19% in their cohort with PTFE and PET grafts, although unfortunately, no differentiation was done between the different materials.

Regarding the comparison of PTFE to PET, only limited and sometimes rather ambiguous data exists.^7^, ^8^, ^9^, ^10^ In a large series of 60 patients receiving CSB as well as subclavian-carotid bypasses, Law et al^7^ described a superior 5-year patency rate of PTFE (95%) compared with PET (84%) and great saphenous vein (65%), showing no statistical significance at all. Accordingly, Owens et al^41^ observed only occlusions of PET grafts constituting an 8-year-patency rate of 78% for PET. However, Wittwer et al^10^ describe no difference in cumulative 5-year patency between PET and PTFE bypasses (85.8% vs 85.7%). On the other hand, Perler et al^8^ and Vitti et al^9^ observed only occlusions of each 3 PTFE grafts in their collectives, constituting a 5-year patency rate for PTFE of 88% and 93%, respectively.

Statements on antithrombotic drug regimens are rare.^10^^,^^17^ In the absence of comorbidities requiring more aggressive antiplatelet or anticoagulant regimens, single platelet inhibition, eg, with acetylsalicylic acid is sufficient^10^ and as such has been prescribed for life in the patients.

With regard to comorbidities and perioperative complications, the described patient collective is comparable to known literature.^8^^,^^9^^,^^19^^,^^40^ In their large collective of 143 supra-aortic reconstructions, Byrne et al^11^ described 2.1% bleeding complications, 0.7% wound infections, and 2,1% neurological complications (such as transient ischemic attack and nonfatal stroke). The collective showed no evidence of neurological symptoms or perfusion impairment at the operated site during the whole follow-up period. The excellent long-term functionality was validated as no differences in occlusion pressure measurements were observed over time or between the respective sides. Postoperatively decreased creatinine and hemoglobin levels are most probably attributable to intraoperative volume administration.

However, this study has several limitations like the retrospective study design, the missing control group of patients receiving PET bypasses or PTFE bypasses with different diameters, and the reduced long-term opportunity for follow-up due to the higher morbidity in TEVAR patients. In addition, the study population remains small and heterogeneous, as patients undergoing bypass for occlusive disease with those requiring debranching before TEVAR were combined, thus, limiting its statistical power. Although the surgical technique is similar, this study could not statistically account for the different underlying pathologies when interpreting patency and complication outcomes. Therefore, further data is needed not only to evaluate specific qualities of different graft materials, but also to compare properties of the materials used as bypass grafts and to account for different pathologies. A clear differentiation between the bypass materials (eg, PTFE and PET) and their specific features—such as diameter and ring-reinforcement in PTFE—is needed to improve comparability and to ensure high-quality data, as the question which graft material actually performs best for SB remains unresolved.^40^ The standardization of graft choices furthermore allows to address additional questions like the optimal coagulation drug of choice or the best surgical technique for particular indications.^10^ The presented data shows promising results for SB creations using ring-reinforced 8 mm PTFE. Further research in form of randomized controlled trials comparing PET to PTFE, as well as investigations of different modifications of these materials to find the optimal material for each patients' individual situation is required.

## Conclusions

The presented findings support the use of ring-reinforced 8 mm PTFE grafts as a reliable option for supra-aortic CSB and CCB, ensuring sustained patency and favorable perioperative outcomes. Future studies are required and should focus on head-to-head comparisons of graft material, diameter, and the presence of ring reinforcement to determine the optimal graft setup for SB surgery.

## Author Contributions

Conception and design: MW, KN, TK, SK, NT, ED, JH.

Analysis and interpretation: MW, NT, ED, MP, NU, GS, JH.

Data collection: MW, TK, SK, GS, JH.

Writing the article: MW, MP, JH.

Critical revision of the article: MW, KN, TK, SK, NT, ED, MP, NU, GS, JH.

Final approval of the article: MW, KN, TK, SK, NT, ED, MP, NU, GS, JH.

Statistical analysis: MW, NU, JH.

Obtained funding: Not applicable.

Overall responsibility: JH.

## Patient consent

All patients provided written informed consent for participation and publication.

## Data availability

The data used and analyzed during the current study are available from the corresponding author per reasonable request.

## Disclosures

None.
